# Socioeconomic and Intersectional Differences in Mathematics Achievement Change: Psychosocial and Contextual Evidence from Chilean Students

**DOI:** 10.3390/bs16101748

**Published:** 2026-09-24

**Authors:** Sonia Salvo-Garrido, Karina Polanco-Levicán, Ana Moraga-Pumarino, José Luis Gálvez-Nieto, Sergio Dominguez-Lara

**Affiliations:** 1 Departamento de Matemática y Estadística, Universidad de La Frontera, Temuco 4780000, Chile; 2 Escuela de Psicología, Facultad de Ciencias Sociales y Educación, Universidad Santo Tomás, Temuco 4800916, Chile; kpolancol@santotomas.cl; 3 Departamento de Administración y Economía, Universidad de La Frontera, Temuco 4780000, Chile; ana.moraga@ufrontera.cl; 4 Departamento de Trabajo Social, Universidad de La Frontera, Temuco 4780000, Chile; jose.galvez@ufrontera.cl; 5 Instituto de Investigación, Facultad de Ciencias de la Comunicación, Turismo y Psicología, Universidad de San Martín de Porres, Lima 15024, Peru; sdominguezl@usmp.pe

**Keywords:** mathematics achievement, academic self-concept, self-efficacy, school violence, socioeconomic status, Indigenous background, intersectionality, longitudinal change

## Abstract

Mathematics achievement is shaped by psychosocial, family, school, and contextual factors, yet less is known about how these influences are associated with longitudinal achievement change and socioeconomic inequalities. This study examined mathematics achievement change between Grade 4 and Grade 8 among 121,109 Chilean students, with particular attention to socioeconomic status (SES), sex, and Indigenous background. Longitudinal SIMCE data linked mathematics scores from Grade 4 in 2015 and Grade 8 in 2019. Three-level linear mixed-effects models were estimated, with repeated measurements nested within students and students nested within their Grade 4 schools. Model comparison was conducted using maximum likelihood, and the final model was re-estimated using restricted maximum likelihood. Academic engagement and self-efficacy, academic self-concept, parental education, cultural capital, parental educational expectations, and classroom order and engagement were positively associated with mathematics achievement, whereas school violence, peer aggression, and perceived discrimination were negatively associated with achievement. Under the linear SES specification, the best-fitting model included a Time × Sex × Indigenous Background × SES interaction, indicating differentiated socioeconomic patterns of longitudinal change. However, a sensitivity analysis treating SES as a five-category factor did not support the omnibus four-way interaction, although robust Time × SES and Time × Sex × SES interactions remained. These findings indicate that socioeconomic differences in mathematics achievement change vary by sex, while evidence for additional moderation by Indigenous background is sensitive to the functional specification of SES.

## 1. Introduction

Mathematics achievement is a central educational and developmental outcome because it is closely related to students’ academic progression, access to advanced learning opportunities, and later educational and occupational pathways (Fu et al., 2025; Katsantonis, 2025). Beyond its cognitive dimension, mathematics achievement is also shaped by psychosocial and contextual processes, including students’ academic self-beliefs, engagement, perceived competence, school safety, peer relations, and experiences of discrimination (Costa et al., 2024). Therefore, understanding mathematics achievement requires attention not only to instructional and socioeconomic conditions, but also to the behavioral, motivational, emotional, and relational contexts in which students learn mathematics (Affuso et al., 2025; Costa et al., 2024).

Longitudinal evidence is particularly important because achievement gaps may change over time rather than remain stable across schooling. Students do not experience mathematics learning as a single isolated outcome; instead, their achievement develops through repeated interactions between individual characteristics, family resources, school environments, and broader social inequalities (Costa et al., 2024; Huang et al., 2025). From a social–ecological perspective, changes in mathematics achievement over time can be considered within the multiple interacting systems that shape students’ opportunities, motivation, emotional security, and engagement with learning (Bronfenbrenner, 1986; Bronfenbrenner & Morris, 2006).

### 1.1. Mathematics Achievement as a Developmental and Psychosocial Outcome

Mathematics achievement is a key indicator of students’ academic development because mathematical competencies are cumulative and provide the foundation for later learning in science, technology, engineering, economics, and everyday problem solving. Difficulties in mathematics during primary and lower secondary education may restrict students’ access to more advanced academic pathways and contribute to long-term educational inequalities (Costa et al., 2024; Katsantonis, 2025). For this reason, understanding how mathematics achievement develops over time remains a central issue in educational psychology, developmental research, and school-based behavioral sciences.

Mathematics achievement, however, should not be understood only as the result of cognitive ability or instructional exposure. Students’ performance in mathematics is also closely related to motivational, emotional, and behavioral processes, including academic engagement, self-efficacy, self-concept, persistence, and perceived control (Affuso et al., 2025; Almeida et al., 2026; Lara et al., 2025). These psychosocial factors may influence how students interpret mathematical challenges, how much effort they invest, and how they respond to errors, difficulties, and academic pressure (Qian, 2025). Recent longitudinal evidence has shown that mathematics self-concept is connected to students’ affective and motivational experiences in mathematics, including mathematics anxiety and mathematics interest (Çelik Demirci et al., 2026). Therefore, mathematics achievement may be associated not only with accumulated learning opportunities, but also with students’ academic self-beliefs and engagement with mathematics (Affuso et al., 2025; Peura et al., 2026).

A longitudinal perspective is particularly relevant because achievement gaps may widen, narrow, or remain stable across schooling (Peura et al., 2026; Salvo-Garrido et al., 2026). Cross-sectional differences in mathematics scores provide important information about inequalities at a given point in time, but they do not show whether differences between groups change as students progress through school. By examining achievement between Grade 4 and Grade 8, it becomes possible to identify whether early differences are maintained or amplified during a period marked by increasing academic demands, greater curricular complexity, and changes in students’ motivational and social experiences.

From a social–ecological perspective, mathematics achievement can be understood within multiple interacting systems. Individual self-beliefs and engagement operate alongside family educational resources, parental expectations, school climate, peer relations, and broader socioeconomic and sociocultural conditions. These factors may contribute to students’ opportunities to learn, their emotional security in school, and their capacity to remain engaged with mathematics over time (Bronfenbrenner, 1986; Bronfenbrenner & Morris, 2006; Salvo-Garrido et al., 2026). Accordingly, the present study conceptualizes mathematics achievement as a developmental and psychosocial outcome embedded in family, school, and social contexts. This conceptualization is also consistent with recent systematic evidence showing that individual, family, school, and contextual factors jointly contribute to understanding academic achievement and educational development over time (Salvo-Garrido et al., 2026).

### 1.2. Academic Self-Beliefs, Engagement, and Mathematics Achievement

Students’ academic self-beliefs are central to understanding mathematics achievement because they influence how students interpret their competence, regulate their effort, and respond to academic difficulty (Almeida et al., 2026; Maamin et al., 2022; Qian, 2025). In mathematics, these processes may be particularly important because learning is cumulative and requires students to build on previously acquired knowledge while engaging with increasingly complex mathematical concepts and tasks. Students who perceive themselves as capable of learning mathematics may be more likely to persist, participate, and use learning opportunities effectively, whereas students with weaker self-beliefs may disengage or avoid mathematically demanding situations (Affuso et al., 2025; Maamin et al., 2022; Peura et al., 2026).

Academic self-concept refers to students’ perceptions of their own competence in a specific academic domain. In mathematics, self-concept is closely related to students’ motivation, interest, anxiety, and achievement (Almeida et al., 2026; Qian, 2025). Recent longitudinal evidence indicates that mathematics self-concept is embedded in broader affective and motivational processes (Çelik Demirci et al., 2026). For example, Çelik Demirci et al. (2026) showed that mathematics interest played a prospective indirect role in the relationship between mathematics anxiety and mathematics self-concept, highlighting the importance of understanding mathematics learning through psychosocial mechanisms. This perspective suggests that mathematics achievement is associated not only with prior achievement and instructional exposure, but also with students’ beliefs about their own ability to succeed in mathematics.

Academic engagement and self-efficacy are also relevant for mathematics achievement. Self-efficacy reflects students’ beliefs about their capacity to organize and carry out the actions required to succeed in academic tasks, whereas engagement captures students’ behavioral, emotional, and cognitive involvement in learning. Students with stronger engagement and self-efficacy may be more likely to invest effort, sustain attention, seek help when needed, and remain involved in mathematics learning despite difficulties (Affuso et al., 2025; Maamin et al., 2022). Previous research has consistently linked self-efficacy, engagement, and academic self-beliefs to mathematics performance and academic development, while longitudinal evidence also shows that family, teacher, and peer support predict different components of school engagement across primary and secondary education (Affuso et al., 2025; Lara et al., 2025; Peura et al., 2026).

These psychosocial factors are especially relevant during the transition from primary to lower secondary education, when mathematics content becomes more abstract and academic demands increase. During this period, students’ beliefs about their competence and their level of engagement may either support continued learning or contribute to disengagement and less favorable mathematics achievement (Affuso et al., 2025; Maamin et al., 2022; Peura et al., 2026). Therefore, examining academic engagement, self-efficacy, and self-concept together provides a more complete understanding of the psychological resources associated with students’ mathematics performance across this educational period (Affuso et al., 2025; Almeida et al., 2026; Çelik Demirci et al., 2026; Lara et al., 2025; Peura et al., 2026).

### 1.3. Family Resources and School Climate

Family resources and school climate are important contextual conditions associated with students’ mathematics achievement. From a social–ecological perspective, students’ academic development is shaped not only by their individual self-beliefs and engagement, but also by the educational, emotional, and relational environments in which learning takes place (Bronfenbrenner, 1986; Bronfenbrenner & Morris, 2006). Family educational resources and expectations may support achievement by providing students with academic guidance, learning materials, structured routines, and motivational messages about the value of schooling (Katsantonis, 2025; Waters et al., 2025). In contrast, limited family resources may restrict access to learning opportunities and contribute to academic disadvantage (Waters et al., 2025).

Parental education, cultural capital, and parental educational expectations are particularly relevant for understanding mathematics achievement and its broader educational context. Parents with higher educational resources may be better positioned to support schoolwork, communicate academic expectations, and navigate educational systems (Waters et al., 2025). Similarly, cultural capital, reflected in the availability of books and learning-related resources at home, may represent broader family educational resources that support students’ academic development (Gubbins & Otero, 2020; Salvo-Garrido et al., 2026). Parental educational expectations may also influence students’ motivation and persistence by communicating beliefs about future educational attainment and academic success (Katsantonis, 2025; Marcenaro-Gutierrez et al., 2025; Qin et al., 2026). Previous research has shown that parental expectations and family educational resources are positively associated with students’ academic achievement and educational trajectories, and that socioeconomic and family-level factors may shape academic development through parental involvement, parenting, and cognitive-developmental pathways (Gubbins & Otero, 2020; Katsantonis, 2025; Marcenaro-Gutierrez et al., 2025; Qin et al., 2026; Waters et al., 2025).

School climate is another key context for mathematics learning. Safe, orderly, and supportive schools may facilitate students’ engagement, concentration, and willingness to participate in academically demanding tasks (Graham et al., 2026; Ortega-Rodríguez, 2025). In contrast, school violence, peer aggression, misconduct, and discrimination may undermine students’ emotional security, sense of belonging, and perceived control (Lanuque et al., 2024; Lucibello et al., 2026). Longitudinal evidence also indicates that negative student–teacher relationships and less positive classroom climates are associated with later traditional bullying and cyberbullying perpetration, whereas positive classroom climate may function as a protective factor (Thornberg et al., 2026).

Recent evidence from Behavioral Sciences supports the relevance of school violence for mathematics-related emotional and cognitive processes. Using TIMSS 2019 data from six countries, including Chile, Kaplan (2026) found that bullying victimization was consistently associated with higher students’ mathematics anxiety and with lower mathematics-related control appraisals in most countries. These findings suggest that peer aggression and unsafe school environments may be linked to mathematics learning not only through academic disruption, but also through affective and cognitive mechanisms.

Perceived discrimination at school may represent an additional contextual risk. Exposure to discrimination or exclusion can weaken students’ sense of belonging and reduce trust in the school environment (Lucibello et al., 2026). For students from socially or culturally marginalized groups, discriminatory experiences may interact with broader inequalities and contribute to less favorable academic outcomes. Therefore, examining family resources, school climate, violence, and discrimination together provides a more comprehensive understanding of the contextual conditions associated with mathematics achievement across educational settings (Lucibello et al., 2026; Bravo-Villa & Mansilla-Sepúlveda, 2026).

### 1.4. Socioeconomic and Intersectional Inequalities in Mathematics Achievement

Socioeconomic status is one of the most consistent predictors of mathematics achievement (Graham et al., 2026; Ng et al., 2021). Students from higher socioeconomic backgrounds often have greater access to educational resources, enriched learning environments, academic support, and schools with stronger organizational and instructional conditions. In contrast, socioeconomic disadvantage may limit access to learning opportunities and expose students to less favorable academic and psychosocial environments (Graham et al., 2026; Huang et al., 2025). These differences may accumulate over time, producing widening gaps in mathematics achievement across schooling (Ng et al., 2021). Longitudinal evidence also suggests that racial and ethnic differences in mathematics difficulties emerge early and are closely intertwined with antecedent, opportunity, and family socioeconomic factors (Ng et al., 2021; Morgan & Hu, 2026).

In Chile, socioeconomic inequalities are closely linked to educational opportunities. The school system has historically been characterized by strong socioeconomic stratification, with students’ academic trajectories shaped by family resources, school composition, contextual conditions, and students’ relative socioeconomic position within the classroom (Murillo et al., 2023; Palma, 2025). Examining SES as a longitudinal predictor of mathematics achievement change is essential for understanding how educational inequalities may differ between educational stages.

Sex is another relevant dimension in mathematics achievement research. Although mathematics has often been socially represented as a male-typed domain, empirical evidence on sex differences in mathematics achievement is mixed and context-dependent, with studies reporting small, variable, or context-specific differences across countries, grade levels, and assessment domains (Bohrnstedt et al., 2024; Leng et al., 2024; Martinot et al., 2025). Rather than assuming a uniform sex gap, it is therefore important to examine whether boys and girls differ in mathematics achievement change over time and whether these differences vary according to other social and contextual conditions.

Indigenous background is also relevant in the Chilean educational context. Previous Chilean evidence has documented achievement differences and differentiated educational trajectories associated with Indigenous background, as well as persistent challenges related to Mapuche educational inclusion, cultural recognition, discrimination, and the place of Indigenous knowledge and language within the school system (Saavedra Vallejos, 2021; Alvarado-Urbina, 2025; Mansilla-Sepúlveda et al., 2025; Bravo-Villa & Mansilla-Sepúlveda, 2026). However, Indigenous background should not be interpreted only as an individual characteristic. It may also reflect broader sociocultural and institutional conditions that shape students’ opportunities to learn, their sense of belonging, and their experiences within school environments.

An intersectional perspective is useful for understanding how these dimensions operate together. Socioeconomic status, sex, and Indigenous background may not be associated with mathematics achievement independently or uniformly. Instead, longitudinal achievement change may differ according to the combined configuration of these social positions. For example, socioeconomic disadvantage may have different implications for boys and girls, and these differences may vary further according to Indigenous background. Examining these intersections makes it possible to identify patterns of inequality that would remain hidden if SES, sex, or Indigenous background were analyzed separately (McCall, 2005).

Accordingly, this study examines mathematics achievement change through an ecological and intersectional framework. This approach recognizes that students’ academic development is embedded within psychosocial, family, school, socioeconomic, and sociocultural contexts. Such an approach is particularly relevant for understanding how mathematics achievement inequalities differ between primary and lower secondary education and across social groups (Bronfenbrenner, 1986; Bronfenbrenner & Morris, 2006; McCall, 2005).

### 1.5. Current Study

Despite growing evidence on the psychosocial and contextual correlates of mathematics achievement, less is known about how socioeconomic differences in achievement change between primary and lower secondary education and whether such longitudinal differences vary jointly by sex and Indigenous background in the Chilean context (Salvo-Garrido et al., 2026).

Building on the above literature, the present study examined mathematics achievement change between Grade 4 and Grade 8 among Chilean students. The study focused on the association of mathematics achievement with psychosocial, family, school, and contextual factors, with particular attention to socioeconomic status, sex, and Indigenous background. By linking national assessment data from Grade 4 in 2015 to Grade 8 in 2019, this study provides longitudinal evidence on mathematics achievement differences between two assessment occasions spanning a four-year educational period.

The study makes three main contributions. First, it examines mathematics achievement as a developmental and psychosocial outcome, incorporating academic engagement, self-efficacy, academic self-concept, family educational resources, school climate, violence, and discrimination. Second, it analyzes whether socioeconomic inequalities in mathematics achievement differ between Grade 4 and Grade 8. Third, it adopts an intersectional perspective by testing whether longitudinal achievement change varies according to the combined configuration of SES, sex, and Indigenous background.

Accordingly, the study was guided by the following hypotheses. Hypothesis 1 (H1) proposed that predictors from multiple ecological domains, including individual, family, school, and contextual factors, would be significantly associated with mathematics achievement. Hypothesis 2 (H2) proposed that students from higher socioeconomic groups would show more favorable mathematics achievement change between Grade 4 and Grade 8. Hypothesis 3 (H3) proposed that mathematics achievement change would differ according to students’ sex and Indigenous background. Hypothesis 4 (H4) proposed that longitudinal mathematics achievement change would vary according to the combined configuration of SES, sex, and Indigenous background between Grade 4 and Grade 8.

Given the longitudinal observational nature of the data, the study does not aim to establish causal effects. Rather, it seeks to identify patterns of association and group differences in mathematics achievement between Grade 4 and Grade 8. In doing so, it contributes to a psychosocial, ecological, and intersectional understanding of how mathematics achievement inequalities may differ across educational stages and social groups within a highly stratified educational context.

## 2. Materials and Methods

### 2.1. Participants

This study used a longitudinal observational design based on secondary data from Chile’s National System for Educational Quality Assessment (SIMCE). The study followed a cohort of students assessed in mathematics in Grade 4 in 2015 and again in Grade 8 in 2019. Student records from both assessment years were linked using anonymized student identifiers, allowing within-student change in mathematics achievement between Grade 4 and Grade 8 to be examined over a four-year period.

The initial Grade 4 mathematics cohort comprised 208,563 students with valid mathematics achievement scores in 2015. Of these, 169,466 students (81.3%) were also observed in Grade 8 in 2019, whereas 39,097 students (18.7%) were not retained longitudinally. Among the students observed at both waves, 121,109 had complete information on all variables included in the final analytical model and therefore constituted the final analytical sample. The sample included 58,236 boys (48.1%) and 62,873 girls (51.9%). Indigenous students represented 15.3% of the sample, whereas 84.7% were non-Indigenous students.

The longitudinal observational design allowed within-student change in mathematics achievement between Grade 4 and Grade 8 to be examined while accounting for the hierarchical structure of the data, with repeated measurements nested within students and students nested within their Grade 4 schools. Baseline psychosocial, family, school, and contextual characteristics were used to examine their associations with mathematics achievement levels and with differential longitudinal change, where interactions with Time were explicitly modeled.

### 2.2. Measures

The study included mathematics achievement scores and a set of questionnaire-based indices measured at baseline in Grade 4. These indices captured individual psychosocial factors, family resources, school climate, and contextual characteristics. A detailed description of the indices, source questionnaires, and interpretation of higher scores is provided in Supplementary Table S1.

#### 2.2.1. Mathematics Achievement

Mathematics achievement was measured using students’ SIMCE mathematics scores obtained in Grade 4 in 2015 and Grade 8 in 2019. SIMCE is Chile’s national standardized assessment system and is administered by the National Educational Quality Assurance agency. The mathematics scores provide comparable measures of students’ achievement at the two assessment occasions and were used to examine longitudinal change between Grade 4 and Grade 8. In the longitudinal dataset, each student contributed two mathematics achievement scores, corresponding to Grade 4 and Grade 8.

#### 2.2.2. Individual Psychosocial Factors

Individual psychosocial factors were derived from the Grade 4 student questionnaire. These variables captured students’ academic self-beliefs, engagement, and perceptions of the school environment, including Academic Engagement and Self-Efficacy, Academic Self-Concept, Teacher Regulation and Normative Guidance, School Safety Perception, and School Violence and Peer Aggression. Higher scores indicated higher levels of the corresponding construct. Additional information on the construction and interpretation of these indices is provided in Supplementary Table S1.

#### 2.2.3. Family Factors

Family factors were derived from the Grade 4 parent/guardian questionnaire. These included Parental Education, Cultural Capital, Parental Educational Expectations, and Perceived Discrimination at School. Parental Education and Cultural Capital were used as indicators of family educational resources. Cultural Capital was operationalized through the availability of books and learning-related resources at home. Parental Educational Expectations captured parents’ aspirations regarding their child’s future educational attainment. Perceived Discrimination at School reflected family-reported experiences of discrimination, exclusion, or unfair treatment within the school context. Higher scores indicated higher levels of the corresponding construct. Additional information on these indices is provided in Supplementary Table S1.

#### 2.2.4. School Climate Factors

School-level factors were derived from Grade 4 school or teacher questionnaire indices. These variables captured organizational and relational aspects of the school environment. Classroom Order and Engagement reflected the extent to which classrooms were perceived as orderly and academically oriented. School Violence Climate captured broader perceptions of violence or conflict within the school context. Teacher Collaboration reflected collaborative practices among teachers. Student Misconduct captured disruptive or inappropriate student behavior within the school environment. Higher scores indicated higher levels of the corresponding construct. Additional information on these indices is provided in Supplementary Table S1.

#### 2.2.5. Contextual and Sociodemographic Variables

Contextual and sociodemographic variables included sex, Indigenous background, socioeconomic status, school dependency, and school location. Sex was coded according to student records and included as a categorical predictor. The variable included binary categories for boys and girls and is therefore interpreted as sex as recorded in the SIMCE database, rather than as a measure of gender identity. Indigenous background was derived from parent/guardian questionnaire information indicating whether the student’s father and/or mother was reported as belonging to one of the Indigenous peoples recognized in Chile. The original questionnaire used yes/no response categories for each parent, and this information was combined to identify a broad indicator of Indigenous family background. Socioeconomic status was measured using the official five-level SIMCE classification, ranging from the lowest to the highest socioeconomic category. In the primary mixed-effects models, SES was treated as an ordinal covariate and centered at the middle category. As a sensitivity analysis, SES was also modeled as a five-category factor to assess whether the substantive conclusions depended on the assumption of a linear socioeconomic gradient. School dependency distinguished public, subsidized private, and private paid schools. School location distinguished urban and rural schools.

### 2.3. Procedure and Ethical Considerations

This study used anonymized secondary data from Chile’s National System for Educational Quality Assessment (SIMCE). SIMCE assessments and questionnaires are administered by the national educational quality assurance agency as part of the official evaluation system. The present study linked Grade 4 mathematics achievement data from 2015 with Grade 8 mathematics achievement data from 2019 using anonymized student identifiers. Baseline questionnaire information from Grade 4 was used to characterize individual psychosocial factors, family resources, school climate, and contextual characteristics.

The research team did not participate in the original data collection process and had no direct contact with students, families, teachers, or schools. The data set used for analysis did not include personally identifiable information. All analyses were conducted on anonymized records and results are reported only in aggregate form. Therefore, individual students, families, teachers, or schools cannot be identified from the reported findings.

The study involving human participants was approved by the Scientific Ethics Committee of the Universidad de La Frontera (Case No. 048_25; Protocol No. 020/25). The data were analyzed in anonymized form, and the research team had no access to personally identifiable information. Because the study relied exclusively on pre-existing anonymized secondary data and involved no direct contact with participants, individual informed consent was not obtained by the research team. The use of Indigenous-background information was limited to aggregate statistical analyses aimed at examining educational inequalities; no individual- or school-level identification was possible. The use of the data was restricted to research purposes and followed the confidentiality principles established for the management of SIMCE information.

### 2.4. Statistical Analysis

Mathematics achievement change between Grade 4 and Grade 8 was analyzed using three-level linear mixed-effects models (LMMs), an approach commonly used for longitudinal and repeated-measures data because it allows within-student change over time and between-student differences in achievement to be estimated simultaneously (Raudenbush & Bryk, 2002; Singer & Willett, 2003). In the present study, mathematics achievement scores measured in Grade 4 and Grade 8 were nested within students, and students were nested within their Grade 4 schools. Random intercepts were therefore specified at both the student and school levels to account for within-student dependence across the two measurement occasions and clustering of students within schools. Grade 4 school membership was used to define the school-level clustering structure because all psychosocial, family, school-climate, and contextual predictors were measured at baseline. Because each student contributed only two repeated observations, the covariance structure was modeled through the student-level random intercept rather than by estimating an additional unstructured residual covariance matrix.

Compared with population-averaged approaches, such as generalized estimating equations, the mixed-effects framework was particularly appropriate because it allowed student- and school-level sources of dependence to be modeled explicitly and their variance components to be estimated. More flexible growth models were not pursued because the availability of only two measurement occasions did not permit the reliable estimation of more complex patterns of change, such as nonlinear growth.

To assess potential selection bias, two complementary analyses were conducted. First, longitudinal attrition was examined among students with valid Grade 4 mathematics scores by comparing those who were also observed at Grade 8 with those who were not retained longitudinally. Second, among students observed at both waves, those included in the final analytical sample were compared with those excluded because of missing covariate information. Baseline mathematics achievement was compared using Welch’s *t*-tests and Cohen’s d, whereas differences in sex, Indigenous background, SES, school dependency, and school location were examined using chi-square tests and Cramér’s V. Of the 208,563 students with Grade 4 mathematics scores, 169,466 (81.3%) were observed again at Grade 8 and 39,097 (18.7%) were not. Students retained longitudinally had higher Grade 4 mathematics achievement than those not retained (M = 265.48 vs. 237.31; Cohen’s d = 0.60), whereas sociodemographic differences were small (Cramér’s V = 0.009–0.083). Among the 169,466 students observed at both waves, 121,109 (71.5%) were included in the final analytical sample, whereas 48,357 (28.5%) were excluded because of missing covariate information. Complete-case inclusion was associated with a smaller baseline achievement difference (M = 268.01 vs. 259.14; Cohen’s d = 0.19), and sociodemographic differences were also small (Cramér’s V = 0.005–0.056). No missing-data imputation was performed; the multivariable models were estimated using complete cases for the variables included in the final model.

No observations were excluded based on outlier detection; exclusions were limited to cases with missing data required for the longitudinal and multivariable analyses.

Time was modeled as a two-level factor representing the two measurement occasions: Grade 4 in 2015 and Grade 8 in 2019. Grade 4 was used as the reference category; therefore, the coefficient for Time represented the expected change in mathematics achievement from Grade 4 to Grade 8, after controlling for the remaining predictors. Socioeconomic status (SES) was coded as an ordinal variable ranging from 1 to 5 and centered at the middle category prior to model estimation (${SES}_{ci}={SES}_{i}-3$). All continuous baseline questionnaire-based predictors were mean-centered prior to model estimation to improve the interpretability of the model intercept. Mean-centering did not alter the estimated slopes, interaction effects, model fit, or predicted marginal means. Thus, the model intercept and lower-order effects involving SES were interpreted at the middle socioeconomic level. To evaluate the sensitivity of the results to the assumption of a linear SES gradient, the final interaction structure was additionally re-estimated with SES specified as a five-category factor. The categorical specification allowed differences across SES levels to be estimated without imposing linearity.

The general specification of the model was:

$$Y_{ijt}=\beta_{0}+\beta_{1}Time_{ijt}+\sum_{k=1}^{p}\beta_{k}X_{kij}+\upsilon_{j}+\;u_{ij}+\varepsilon_{ijt}$$

where $Y_{ijt}$ represents the mathematics achievement score of student *i* in the school *j* at measurement occasion t; $\beta_{0}$ is the fixed intercept; $\beta_{1}$ represents the fixed effect of time; $X_{kij}$ represents the set of individuals, family, school, and contextual predictors measured in Grade 4; $\beta_{k}$ represents the corresponding fixed-effect coefficients; $\upsilon_{j}$ is the school-specific random intercept capturing unobserved between-school variability; $u_{ij}$ is the student-specific random intercept capturing unobserved between-student variability within schools; and $\varepsilon_{ijt}$ is the residual error term.

The modeling strategy proceeded in several steps. First, a null model was estimated to provide a baseline for model comparison. Second, an integrated model including individual psychosocial factors, family factors, school climate factors, and contextual characteristics was estimated. Third, a series of interaction models was estimated to examine whether longitudinal mathematics achievement change varied by sex, Indigenous background, and SES. These models included two-way interactions between Time and each focal contextual variable, a three-way interaction model including Time × Sex × Indigenous Background, and a four-way interaction model including Time × Sex × Indigenous Background × SES_c. All models included random intercepts for students and Grade 4 schools, thereby maintaining the same hierarchical structure across model comparisons.

Model comparison was conducted using maximum likelihood (ML) estimation, which is appropriate when comparing models that differ in fixed-effect structure. Competing models were compared using the −2 log-likelihood, Akaike Information Criterion (AIC), and Bayesian Information Criterion (BIC), with lower values indicating better model fit (Akaike, 1974; Schwarz, 1978; Burnham & Anderson, 2002). For nested models, likelihood-ratio tests were additionally used to evaluate whether the inclusion of higher-order interaction terms significantly improved model fit.

Under the primary linear SES specification, the final model included the four-way interaction among Time, Sex, Indigenous Background, and centered SES:

$$Y_{ijt}=\beta_{0}+\beta_{1}Time_{ijt}+\sum_{k=1}^{p}\beta_{k}X_{kij}+\beta_{p+1}\left(Time_{ijt}\times Sex_{i}\times {Indigenous\;Background}_{i}\times SES_{ci}\right)+\upsilon_{j}+u_{ij}+\varepsilon_{ijt}$$

where ${SES}_{ci}$ represents socioeconomic status for student (*i*), centered at the middle category, with ${SES}_{ci}={SES}_{i}-3$. The final model included all corresponding lower-order terms required for the correct interpretation of the four-way interaction. This specification allowed us to examine whether longitudinal mathematics achievement change between Grade 4 and Grade 8 varied according to the combined configuration of socioeconomic status, sex, and Indigenous background, while adjusting for individual psychosocial, family, school climate, and contextual factors.

All corresponding lower-order terms associated with the four-way interaction were included in the model. Degrees of freedom and significance tests for fixed effects were obtained using the Satterthwaite approximation. Predicted marginal means were estimated from the final REML model to facilitate interpretation of the significant interactions. Simple effects and simple slopes were examined using estimated marginal means and trend contrasts, with 95% confidence intervals; Holm-adjusted *p*-values were used where multiple post hoc contrasts were evaluated. For the large-sample marginal-mean analyses, asymptotic inference was used. Marginal and conditional R^2^ values were calculated using Nakagawa’s approach (Nakagawa et al., 2017) to quantify the variance explained by the fixed effects and by the complete mixed model, respectively. Analyses were conducted in RStudio (version 2026.04.0+526; Posit Software, PBC, Boston, MA, USA) using the lme4, lmerTest, emmeans, dplyr, tidyr, and performance packages (Bates et al., 2015; Kuznetsova et al., 2017; Lenth & Piaskowski, 2026).

The statistical analysis code used to estimate the models, conduct model comparisons, and obtain predicted marginal means can be made available by the corresponding author upon reasonable request. The student-level SIMCE data used in this study are not publicly available due to confidentiality restrictions associated with the use of anonymized national assessment records.

## 3. Results

### 3.1. Descriptive Statistics

Table 1 summarizes the descriptive characteristics of the analytical sample. Because the sociodemographic composition of the sample was reported in the Participants section, this section focuses on the distribution of the study variables included in the analytical models. Mathematics achievement scores were relatively stable across the two assessment points, with a mean of 268.0 points in Grade 4 and 269.5 points in Grade 8. However, variability was substantial at both time points, indicating considerable heterogeneity in students’ mathematics achievement and longitudinal change.

Among the individual psychosocial predictors, students reported relatively high levels of Academic Engagement and Self-Efficacy, Academic Self-Concept, Teacher Regulation and Normative Guidance, and School Safety Perception, whereas School Violence and Peer Aggression showed lower average levels. Family-level indicators showed moderate levels of parental education and cultural capital, high parental educational expectations, and low average levels of perceived discrimination at school. School climate indicators also suggested relatively high classroom order and engagement, moderate teacher collaboration, and lower average levels of school violence climate and student misconduct.

Socioeconomic status reflected the unequal distribution observed in the Chilean school system. Students were distributed across the five ordered SES categories as follows: lowest = 52.0%, low = 25.4%, middle = 8.6%, high = 7.3%, and highest = 6.7%. Given its ordered structure, SES was included in the primary mixed-effects models as an ordinal covariate centered at the middle category, with a complementary sensitivity analysis treating SES as a five-category factor. These descriptive results provide the basis for the subsequent linear mixed models, which estimated within-student change between Grade 4 and Grade 8 while adjusting for individual psychosocial, family, school climate, and contextual factors.

### 3.2. Model Comparison and Selection

A series of three-level linear mixed models was estimated using maximum likelihood (ML) estimation to identify the specification that best represented students’ longitudinal mathematics achievement change. Table 2 presents the −2 log-likelihood, AIC, and BIC values for the estimated models. All models included random intercepts for students and Grade 4 schools.

The null model showed the poorest fit, whereas the integrated model substantially improved model fit by incorporating individual psychosocial factors, family resources, school climate factors, and contextual characteristics. This improvement indicated that mathematics achievement levels and longitudinal change were better represented after accounting for predictors operating across multiple ecological domains.

The inclusion of interaction terms provided additional improvements in model fit, suggesting that changes in mathematics achievement between Grade 4 and Grade 8 were not uniform across student groups. Among the two-way interaction models, the model including Time × SES_c showed the greatest improvement. However, under the primary linear SES specification, the four-way interaction model including Time × Sex × Indigenous Background × SES_c showed the lowest −2 log-likelihood, AIC, and BIC values (−2LL = 2,422,212; AIC = 2,422,282; BIC = 2,422,646).

This four-way interaction model improved fit relative to the three-way Time × Sex × Indigenous Background model (−2LL = 2,423,169; AIC = 2,423,225; BIC = 2,423,516) and the Time × SES_c model (−2LL = 2,422,672; AIC = 2,422,722; BIC = 2,422,982). Accordingly, the four-way interaction model was selected as the final model and re-estimated using restricted maximum likelihood (REML) for the interpretation of fixed effects.

### 3.3. Fixed Effects of the Final Model

After model selection, the final four-way interaction model was re-estimated using restricted maximum likelihood (REML). Type III tests of fixed effects were used to evaluate the omnibus significance of the fixed effects and interaction terms, whereas Table 3 presents selected fixed-effect estimates from the final REML model, including the main fixed effects and selected conditional interaction estimates relevant to the interpretation of the retained four-way interaction structure. Because the final model included the higher-order interaction among Time, Sex, Indigenous Background, and SES_c, all lower-order terms required by the interaction hierarchy were retained in the model. For parsimony, not all lower-order conditional interaction estimates are displayed in Table 3, although all lower-order terms required by the interaction hierarchy were retained in the model. SES_c was modeled as a centered ordinal covariate; therefore, coefficients involving SES_c should be interpreted as changes associated with a one-unit increase in socioeconomic status relative to the centered middle category, rather than as comparisons among separate SES groups. The model additionally included random intercepts for students and Grade 4 schools.

Type III tests of fixed effects supported the selected four-way interaction model under the primary linear SES specification. The highest-order interaction, Time × Sex × Indigenous Background × SES_c, was statistically significant, F(1, 121,101) = 6.604, *p* = 0.010, indicating that the association between time and mathematics achievement varied across sex and Indigenous-background groups as a function of centered socioeconomic status. Significant lower-order interactions were also observed for Time × Sex, F(1, 121,101) = 91.458, *p* < 0.001; Time × SES_c, F(1, 121,101) = 216.586, *p* < 0.001; Time × Sex × Indigenous Background, F(1, 121,101) = 5.280, *p* = 0.022; and Time × Sex × SES_c, F(1, 121,101) = 4.312, *p* = 0.038. Other lower-order interaction terms were retained to preserve the hierarchical structure of the model.

As shown in Table 3, because the final model retained a Time × Sex × Indigenous Background × SES_c interaction under the primary linear SES specification, the coefficients for Time, Sex, Indigenous Background, SES_c, and their interaction terms should be interpreted as conditional contrasts relative to the reference categories and to SES_c = 0, rather than as average effects across the full sample. The lower-order coefficient for Indigenous Background was not statistically significant after controlling for the remaining predictors and interaction terms; however, Indigenous Background was retained because it formed part of the theoretically relevant higher-order interaction structure. Similarly, the lower-order coefficient for SES_c should not be interpreted as the overall socioeconomic association because its meaning is conditional on the reference categories and the higher-order interaction structure. School dependency and school location were also significantly associated with mathematics achievement, indicating differences in estimated mathematics scores according to school sector and habitat.

Individual psychosocial factors were consistently associated with mathematics achievement. Academic Engagement and Self-Efficacy and Academic Self-Concept showed positive associations with achievement, indicating that students who reported stronger academic engagement, self-efficacy, and academic self-beliefs in Grade 4 tended to obtain higher mathematics scores across the two assessment occasions. Teacher Regulation and Normative Guidance and School Safety Perception also showed positive associations with achievement. In contrast, the School Violence and Peer Aggression index was negatively associated with mathematics achievement, suggesting that adverse peer and school experiences were linked to lower mathematics performance.

Within the family domain, Parental Education, Cultural Capital, and Parental Educational Expectations were positively associated with mathematics achievement, indicating that family educational resources and expectations were linked to higher mathematics scores. Conversely, Perceived Discrimination at School was negatively associated with achievement, suggesting that discriminatory experiences may represent a contextual risk for students’ academic development.

At the school climate level, Classroom Order and Engagement was positively associated with mathematics achievement, whereas School Violence Climate was negatively associated with achievement. Teacher Collaboration and Student Misconduct were not statistically significant after accounting for the school-level random intercept and the remaining predictors in the model. These results suggest that classroom order and engagement and lower levels of school violence are relevant to mathematics achievement, whereas the independent associations of teacher collaboration and student misconduct were not supported in the fully adjusted three-level model.

Overall, the fixed effects supported the psychosocial, ecological, and intersectional framework of the study. Mathematics achievement and longitudinal change were associated with individual self-beliefs and engagement, family educational resources, school climate, and contextual characteristics. At the same time, the significant four-way interaction under the primary linear SES specification indicated that changes in mathematics achievement between Grade 4 and Grade 8 were not uniform but varied according to sex and Indigenous background as a function of centered socioeconomic status. However, this higher-order interaction was sensitive to the functional specification of SES, as the corresponding omnibus interaction was not significant when SES was treated categorically. Accordingly, patterns of longitudinal change are interpreted primarily through the predicted marginal means in Figure 1 and Figure 2 and the sensitivity analyses reported below.

The conditional Time coefficient of 2.35 points corresponded to approximately 0.05 SD of the mathematics achievement score, indicating a small overall change in substantive terms. Standardized coefficients indicated that the largest psychosocial and family associations with mathematics achievement were observed for Academic Engagement and Self-Efficacy (β = 0.13), Parental Educational Expectations (β = 0.10), Academic Self-Concept (β = 0.09), School Violence and Peer Aggression (β = −0.09), and Parental Education (β = 0.08). In contrast, the standardized association for School Safety Perception was negligible (β = 0.006), while Teacher Collaboration (β = −0.007) and Student Misconduct (β = 0.003) were both negligible and not statistically significant in the final three-level model. These results underscore the importance of distinguishing statistical significance from substantive magnitude in this large sample.

At the model level, the final three-level model showed substantial variance at both the student and school levels. The estimated variance components were 805.36 at the student level, 224.23 at the Grade 4 school level, and 679.41 at the residual level, corresponding to approximately 47.1%, 13.1%, and 39.8% of the total variance, respectively. The school-level intraclass correlation coefficient (ICC) was 0.131, indicating that 13.1% of the total variance in mathematics achievement was attributable to differences between Grade 4 schools. The correlation between repeated mathematics achievement observations within the same student was 0.602, reflecting the combined student- and school-level random-intercept variance. The model yielded a marginal R^2^ of 0.220 and a conditional R^2^ of 0.690, indicating that the fixed effects accounted for 22% of the variance in mathematics achievement, whereas the full model, including student- and school-level random effects, accounted for 69% of the variance.

### 3.4. Predicted Longitudinal Mathematics Achievement Change

To facilitate interpretation of the final interaction model, predicted marginal means were estimated from the final REML model. Although SES was modeled as an ordinal covariate rather than as a categorical factor, predictions were obtained at each observed SES value (1–5) to display the estimated socioeconomic gradient in mathematics achievement change. Figure 1 presents adjusted mathematics achievement change from Grade 4 to Grade 8 across these five observed SES values.

As shown in Figure 1, mathematics achievement changes varied substantially according to students’ socioeconomic background. Students in the lowest SES group showed a slight decline in predicted mathematics achievement, with estimated scores of 267.2 in Grade 4 and 266.3 in Grade 8. In contrast, students in higher SES groups showed progressively larger gains over time. Predicted scores increased from 267.9 to 269.5 in SES 2, from 268.5 to 272.7 in SES 3, from 269.2 to 275.9 in SES 4, and from 269.8 to 279.1 in SES 5.

This pattern indicates that socioeconomic inequalities were not limited to differences in initial achievement levels but were also associated with differential achievement change across the observed period. The achievement gap between the lowest and highest SES groups widened from approximately 2.6 points in Grade 4 to 12.8 points in Grade 8. Thus, students from higher socioeconomic backgrounds showed more favorable longitudinal mathematics achievement change between Grade 4 and Grade 8, whereas students from the lowest SES group showed a slight decline.

Figure 2 presents predicted longitudinal mathematics achievement change for selected combinations of sex, Indigenous background, and SES.

Figure 2 illustrates the predicted patterns of longitudinal mathematics achievement change by sex and Indigenous background at the lowest and highest SES levels. Overall, the figure shows contrasting patterns across socioeconomic levels, with less favorable change at SES 1 and more pronounced gains at SES 5, particularly among boys. Differences by Indigenous background were comparatively modest and should be interpreted cautiously in light of the sensitivity analyses.

Among students in SES 1, longitudinal changes were relatively small but differed by sex. Boys showed small gains between Grade 4 and Grade 8, increasing from 269.7 to 270.9 among Indigenous boys and from 270.4 to 271.7 among non-Indigenous boys. In contrast, girls in SES 1 showed lower predicted scores in Grade 8 than in Grade 4, from 264.0 to 261.3 among Indigenous girls and from 264.7 to 261.4 among non-Indigenous girls. Indigenous girls had the lowest predicted Grade 8 mathematics achievement within the lowest SES group.

A different pattern emerged among students in SES 5. In the highest socioeconomic group, all groups showed increases in predicted mathematics achievement, but the magnitude of change differed by sex and Indigenous background. Indigenous boys increased from 273.2 to 286.9, and non-Indigenous boys increased from 271.7 to 283.6. Girls also showed gains, although these were smaller: Indigenous girls increased from 269.0 to 272.4, whereas non-Indigenous girls increased from 265.4 to 273.4.

Simple-slope analyses further clarified the interaction pattern. Among non-Indigenous girls, the estimated socioeconomic gradient in mathematics achievement increased from 0.19 points per SES unit in Grade 4 (95% CI [−0.15, 0.54]) to 3.00 points in Grade 8 (95% CI [2.66, 3.35]), corresponding to an increase in the SES slope of 2.81 points. Among non-Indigenous boys, the SES slope increased from 0.33 (95% CI [−0.02, 0.68]) to 2.97 (95% CI [2.62, 3.33]), an increase of 2.64 points. For Indigenous girls, the SES gradient increased from 1.24 (95% CI [0.26, 2.22]) to 2.77 (95% CI [1.79, 3.75]), whereas for Indigenous boys it increased from 0.87 (95% CI [−0.13, 1.88]) to 4.00 (95% CI [2.99, 5.00]). The corresponding increase in the SES slope was 1.53 points for Indigenous girls and 3.12 points for Indigenous boys. The four-way difference in these longitudinal SES-slope changes was 1.76 points (SE = 0.69, 95% CI [0.42, 3.11], *p* = 0.010), although this higher-order interaction was not robust when SES was modeled categorically. Holm-adjusted post hoc contrasts showed statistically significant Grade 4-to-Grade 8 changes for 17 of the 20 evaluated sex × Indigenous-background × SES combinations, whereas the changes for Indigenous girls at SES 3, SES 4, and SES 5 were not statistically significant after adjustment.

Overall, the predicted patterns of longitudinal change support the interpretation that mathematics achievement change was associated with the combined configuration of SES, sex, and Indigenous background under the primary linear SES specification. The four-way interaction indicates that the pattern of change varied across combinations of these characteristics; however, this higher-order interaction was not supported when SES was modeled categorically. Instead, the most robust pattern across specifications was the differential socioeconomic change over time, particularly by sex, whereas additional moderation by Indigenous background should be interpreted cautiously.

### 3.5. Sensitivity Analysis: SES as a Categorical Variable

To assess the robustness of the results to the functional specification of socioeconomic status, the final model was re-estimated treating SES as a five-category factor rather than as a centered ordinal covariate. The categorical SES model showed a slightly lower −2 log-likelihood and AIC than the linear SES model (−2LL = 2,422,148 vs. 2,422,212; AIC = 2,422,266 vs. 2,422,282), although the BIC favored the more parsimonious linear specification (2,422,879 vs. 2,422,646). A likelihood-ratio test indicated that the categorical specification provided a statistically significant improvement in fit, χ^2^(24) = 64.27, *p* < 0.001.

Importantly, however, the omnibus Time × Sex × Indigenous Background × SES interaction was not statistically significant when SES was modeled categorically, χ^2^(4) = 7.70, *p* = 0.103. In contrast, the Time × SES interaction remained significant, F = 53.17, *p* < 0.001, as did the Time × Sex × SES interaction, F = 4.41, *p* = 0.001. The Time × Sex × Indigenous Background interaction was not significant under the categorical SES specification, F = 0.31, *p* = 0.820. These findings indicate that the evidence for socioeconomic differences in longitudinal mathematics achievement change, and particularly their variation by sex, was robust to the specification of SES, whereas the additional four-way moderation involving Indigenous background was sensitive to whether SES was modeled linearly or categorically.

## 4. Discussion

This study examined mathematics achievement change between Grade 4 and Grade 8 among Chilean students, considering individual psychosocial factors, family resources, school climate, and contextual characteristics. Four hypotheses guided the analyses. First, we expected that predictors from multiple ecological domains would be associated with mathematics achievement (H1). Second, we expected that students from higher socioeconomic groups would show more favorable longitudinal mathematics achievement change (H2). Third, we expected mathematics achievement change to differ according to students’ sex and Indigenous background (H3). Fourth, we expected longitudinal mathematics achievement change to vary according to the combined configuration of socioeconomic status, sex, and Indigenous background between Grade 4 and Grade 8 (H4).

Overall, the findings supported the psychosocial, ecological, and intersectional framework of the study. Mathematics achievement was associated with individual self-beliefs and engagement, family educational resources, school climate, and contextual characteristics, supporting H1. Socioeconomic status emerged as a major source of differentiation in longitudinal achievement change, with students from higher SES groups showing more favorable change between Grade 4 and Grade 8, supporting H2. Evidence for H3 was more nuanced: patterns of longitudinal change differed clearly by sex, whereas the contribution of Indigenous background depended on its joint configuration with sex and SES and on the functional specification of SES. Finally, H4 received partial and specification-dependent support. Under the primary linear SES specification, the final model included a significant Time × Sex × Indigenous Background × SES interaction; however, this four-way interaction was not supported when SES was modeled categorically. Across both specifications, the most robust findings were the Time × SES and Time × Sex × SES interactions, indicating that socioeconomic differences in longitudinal mathematics achievement change varied particularly by sex.

### 4.1. Socioeconomic Differences in Longitudinal Mathematics Achievement Change

Consistent with H2, socioeconomic status was a central source of differentiation in mathematics achievement change between Grade 4 and Grade 8. Students from the lowest SES group showed a slight decline in predicted mathematics achievement, whereas students from progressively higher SES groups exhibited increasingly favorable changes over time. This pattern indicates that socioeconomic inequalities were not limited to differences in initial achievement levels but were also associated with differential longitudinal change across the observed period.

This result is consistent with previous evidence showing that socioeconomic status is one of the most robust predictors of mathematics achievement and academic development (Graham et al., 2026; Ng et al., 2021). Socioeconomic advantages may be associated with greater access to educational resources, stronger family support, more stable learning environments, and schools with better organizational and instructional conditions. Conversely, socioeconomic disadvantage may be associated with more restricted learning opportunities and greater exposure to less favorable academic, emotional, and contextual conditions (Huang et al., 2025; Lucibello et al., 2026). Over time, these differences may contribute to cumulative patterns of advantage and disadvantage.

From a psychosocial perspective, socioeconomic inequalities may also be associated with mathematics achievement through students’ motivational and emotional experiences. Students from more advantaged backgrounds may have greater access to academic support, learning materials, positive feedback, and school environments that reinforce confidence and engagement. In contrast, students from lower socioeconomic backgrounds may be more exposed to academic stress, school instability, discrimination, violence, or reduced expectations, all of which may be associated with lower perceived competence and engagement with mathematics. Therefore, SES may be linked to longitudinal mathematics achievement change not only through material resources, but also through psychosocial opportunities and constraints.

The Chilean context is particularly relevant for interpreting this finding. Latin America and the Caribbean show high levels of socioeconomic school segregation, and Chile has historically exhibited strong links between socioeconomic background, school composition, and educational outcomes (Murillo et al., 2023; Palma, 2025). The present findings indicate that these structural inequalities are expressed not only in cross-sectional differences in achievement, but also in longitudinal mathematics achievement change between Grade 4 and Grade 8. Students from more advantaged socioeconomic backgrounds did not merely start from a more favorable position; they also showed larger increases in predicted mathematics achievement across the observed period.

The widening socioeconomic differences observed between Grade 4 and Grade 8 have important implications for educational equity, although they should be interpreted considering the study’s observational design and patterns of selective attrition. Students retained longitudinally had higher Grade 4 mathematics achievement than those not retained, indicating that attrition was not random with respect to baseline achievement. Accordingly, the magnitude of the observed socioeconomic widening may be affected by selective panel retention. Nevertheless, the consistent Time × SES association across both the linear and categorical SES specifications indicates that socioeconomic differences in mathematics achievement change were robust to the functional form used to represent SES. These findings underscore the importance of examining socioeconomic inequalities early in the educational pathway and of evaluating, in future intervention research, whether sustained academic and psychosocial support can help reduce their persistence or expansion across schooling.

### 4.2. Individual and Family Predictors of Mathematics Achievement

Consistent with H1, mathematics achievement was associated with individual psychosocial factors and family resources. Among the individual predictors, Academic Engagement and Self-Efficacy and Academic Self-Concept showed positive associations with mathematics achievement. These associations were also among the largest standardized effects observed for the questionnaire-based predictors, particularly for Academic Engagement and Self-Efficacy and Academic Self-Concept. These findings are consistent with previous research indicating that students’ academic self-beliefs, motivation, and engagement are important psychosocial resources for mathematics learning (Affuso et al., 2025; Almeida et al., 2026; Maamin et al., 2022; Peura et al., 2026).

The positive association between Academic Self-Concept and mathematics achievement is particularly relevant because mathematics learning involves cumulative knowledge, increasing task complexity, and frequent experiences of challenge or error. Students who perceive themselves as academically capable may be more likely to persist, participate, and recover from setbacks when facing demanding mathematical tasks. This interpretation is consistent with recent longitudinal evidence showing that mathematics self-concept is closely connected to students’ affective and motivational experiences in mathematics, including mathematics anxiety and interest (Çelik Demirci et al., 2026). Thus, the positive association observed in the present study supports the view that students’ self-beliefs constitute relevant psychosocial resources associated with mathematics achievement across the two assessment occasions.

Academic Engagement and Self-Efficacy also emerged as strong positive predictors of mathematics achievement. Students with higher engagement and stronger self-efficacy may be better positioned to sustain effort, regulate their learning, and remain involved in mathematics despite difficulties. This finding aligns with recent evidence from Behavioral Sciences showing that mathematics self-efficacy and metacognitive self-regulation are positively associated with mathematics performance (Katsantonis, 2026). From this perspective, the observed association suggests that students’ engagement, confidence, and self-regulatory resources are meaningfully related to mathematics achievement across the two assessment occasions, rather than providing direct evidence of differential longitudinal change.

Family resources were also significantly associated with mathematics achievement. Parental Education, Cultural Capital, and Parental Educational Expectations showed positive associations with achievement. These findings suggest that family educational resources and expectations may support students’ mathematics learning by providing learning materials, academic guidance, structured routines, and motivational messages about the value of schooling. Previous research has shown that parental expectations and family educational resources are positively associated with students’ academic achievement and broader educational development (Fu et al., 2025; Katsantonis, 2025; Qin et al., 2026).

Conversely, Perceived Discrimination at School was negatively associated with mathematics achievement. This finding suggests that family-reported experiences of discrimination or exclusion may represent a contextual risk for students’ academic development. Discrimination may be associated with a weaker sense of belonging, lower emotional security, and reduced trust in school institutions, which in turn may limit students’ engagement with learning. This is particularly relevant in contexts where socioeconomic and sociocultural inequalities are intertwined with school experiences.

Overall, the associations observed for individual and family predictors support a psychosocial and ecological understanding of mathematics achievement. Achievement levels across the two assessment occasions were associated not only with socioeconomic position and school characteristics, but also with academic self-beliefs, engagement, family educational resources, parental expectations, and experiences of discrimination. These findings reinforce the importance of considering both psychological resources and family contexts when examining mathematics achievement in longitudinal educational settings.

### 4.3. School Climate, Violence, and Contextual Risk

School climate and school violence also emerged as relevant factors associated with mathematics achievement, providing further support for H1. Positive school conditions, such as School Safety Perception and Classroom Order and Engagement, were associated with higher mathematics achievement, whereas School Violence and Peer Aggression, School Violence Climate, and Perceived Discrimination at School were negatively associated with achievement. Teacher Collaboration and Student Misconduct were not statistically significant after accounting for the Grade 4 school-level random intercept and the remaining predictors in the final three-level model. These findings suggest that students’ mathematics achievement was associated not only with individual and family resources, but also with the relational, emotional, and organizational conditions of the school environment.

The negative association between school violence and mathematics achievement is particularly important. Mathematics learning requires sustained attention, persistence, emotional regulation, and willingness to engage with challenging tasks. Violent, disruptive, or unsafe school environments may be associated with reduced opportunities for concentration, participation, and persistence in academic activities, which is consistent with previous evidence linking bullying and peer victimization to poorer academic and socioemotional outcomes (Lanuque et al., 2024; Ortega-Rodríguez, 2025). In the present study, School Violence and Peer Aggression showed one of the larger standardized associations among the questionnaire-based predictors (β = −0.09), indicating that its relationship with mathematics achievement was not only statistically detectable but also comparatively meaningful within the set of measured psychosocial and school-context variables.

This interpretation is consistent with recent cross-cultural evidence showing that bullying victimization is detrimental to mathematics-related emotional and cognitive processes. Kaplan (2026), using TIMSS 2019 data from Chile and five other countries, found that bullying increased students’ mathematics anxiety and reduced perceived control in most contexts. In line with this evidence, the negative associations observed in the present study for School Violence and Peer Aggression and School Violence Climate are consistent with the possibility that adverse school environments are linked to lower mathematics achievement through students’ emotional security, perceived control, and engagement with academic tasks. By contrast, Student Misconduct was not independently associated with mathematics achievement after accounting for school-level clustering and the remaining predictors in the final model.

Perceived Discrimination at School was also negatively associated with mathematics achievement. This finding reinforces the need to understand academic development within broader social and cultural contexts. Discriminatory experiences may be associated with a weaker sense of belonging, lower trust in school institutions, and reduced emotional safety, which may in turn constrain students’ engagement with learning. In contexts marked by ethnic and socioeconomic inequalities, perceived discrimination may therefore represent an additional source of educational disadvantage. Its standardized association with mathematics achievement was modest but negative (β = −0.05), indicating that the relationship was smaller than that observed for School Violence and Peer Aggression but remained substantively relevant within the broader psychosocial context.

The positive association observed for Classroom Order and Engagement suggests that structured and academically oriented classroom environments are meaningfully related to mathematics achievement. Orderly classrooms may provide conditions that facilitate instructional time, reduce disruptions, and support students’ sustained attention to academic tasks. Although Teacher Collaboration was conceptually relevant within the school-climate domain, its independent association with mathematics achievement was not statistically significant in the final three-level model after accounting for school-level clustering and the remaining predictors. This distinction suggests that some school-climate characteristics may operate partly through broader shared school conditions rather than through independent associations detectable once between-school variability is explicitly modeled.

Overall, these findings indicate that school climate is not peripheral to mathematics achievement. Safe, orderly, and inclusive environments were associated with more favorable mathematics achievement, whereas school violence, peer aggression, and perceived discrimination were associated with lower achievement. However, not all school-climate indicators showed independent associations once Grade 4 school-level clustering and the remaining predictors were considered, as illustrated by the nonsignificant estimates for Teacher Collaboration and Student Misconduct. From a psychosocial and ecological perspective, these findings support the relevance of school context for understanding differences in mathematics achievement levels across the two assessment occasions, while avoiding the inference that these baseline school-climate measures directly explained longitudinal change.

### 4.4. Intersectional Differences by Sex, Indigenous Background, and SES

The findings provided partial and specification-dependent support for H4. Under the primary linear SES specification, longitudinal mathematics achievement change differed according to the combined configuration of sex, Indigenous background, and SES. Although Indigenous background was not significant as an isolated main effect after controlling for the remaining predictors and interaction terms, this lower-order coefficient was conditional on the reference categories and SES_c = 0 and therefore should not be interpreted as evidence of an absence of Indigenous-related differences across the full sample. Under the linear SES specification, the significant four-way interaction among Time, Sex, Indigenous Background, and SES indicated that patterns of longitudinal change differed across combinations of these characteristics. However, this interaction was not statistically supported when SES was modeled categorically, indicating that the additional moderation involving Indigenous background was sensitive to the functional specification of SES. Accordingly, the intersectional interpretation is supported most clearly for the joint patterning of socioeconomic status and sex, whereas evidence for further differentiation by Indigenous background should be interpreted cautiously.

Sex differences in mathematics achievement have been widely studied, but prior evidence remains mixed (Bohrnstedt et al., 2024; Leng et al., 2024; Martinot et al., 2025). Some studies report small or context-dependent differences between boys and girls, whereas others identify advantages favoring one group depending on the country, grade level, mathematical domain assessed, and students’ motivational and emotional resources. The present findings are consistent with this more nuanced view. Rather than suggesting a uniform sex gap, the results indicate that patterns of longitudinal mathematics achievement change differed by sex and varied further across socioeconomic levels, while additional differentiation by Indigenous background was less robust across model specifications.

At the lowest socioeconomic level, the predicted patterns showed relatively small Grade 4-to-Grade 8 changes, but these differed by sex. Boys showed slight increases, whereas girls showed declines in predicted mathematics achievement. Within this low-SES context, Indigenous and non-Indigenous students displayed broadly similar sex-specific patterns, suggesting that the clearest differentiation was by sex rather than by Indigenous background alone. Indigenous girls had the lowest predicted Grade 8 score within SES 1, but this descriptive pattern should not be interpreted as evidence of an isolated Indigenous-background effect, particularly given the lack of robustness of the four-way interaction under the categorical SES specification.

A different pattern emerged among students in the highest SES group. In SES 5, all groups showed increases in predicted mathematics achievement, but gains were larger among boys than among girls. Indigenous and non-Indigenous boys showed the strongest increases, whereas girls’ gains were more moderate. These findings reinforce the robustness of the socioeconomic-by-sex pattern in longitudinal achievement change. Although predicted values also differed by Indigenous background within some subgroup combinations, these differences should be interpreted cautiously because the four-way interaction was not supported when SES was modeled categorically.

In the Chilean context, this result is particularly relevant. Indigenous students, particularly Mapuche students, may experience educational conditions shaped by socioeconomic inequality, cultural exclusion, discrimination, and limited recognition of Indigenous knowledge and language within the school system (Bravo-Villa & Mansilla-Sepúlveda, 2026; Mansilla-Sepúlveda et al., 2025; Saavedra Vallejos, 2021). Accordingly, Indigenous background should not be interpreted as an isolated individual attribute or as a direct causal determinant of mathematics achievement change. Rather, it may reflect broader sociocultural and institutional conditions that intersect with socioeconomic position and sex and that are relevant to students’ educational experiences. At the same time, the dichotomous Indigenous-background indicator used in this study does not capture the diversity of Indigenous identities, languages, cultural affiliations, or school experiences in Chile, which limits the specificity of the intersectional interpretation.

Overall, these findings support a cautious intersectional interpretation of longitudinal mathematics achievement change. Socioeconomic status was the most consistent source of differentiation, and its association with change varied robustly by sex across both the linear and categorical SES specifications. Evidence for additional differentiation by Indigenous background was more limited and depended on the functional specification of SES. Thus, the results suggest that educational inequalities are better understood through the joint configuration of social positions rather than through any single dimension considered in isolation. At the same time, the present findings indicate that the most stable intersectional pattern involved socioeconomic status and sex, whereas conclusions involving Indigenous background require greater caution and further investigation.

### 4.5. Educational Implications

The findings have several implications for educational policy and practice. First, the widening socioeconomic differences in mathematics achievement between Grade 4 and Grade 8 highlight the importance of sustained attention to socioeconomic inequalities across the school years. Support provided only in later stages of schooling may be insufficient if these inequalities have already widened earlier in the educational pathway. The present findings therefore identify early socioeconomic disparities in mathematics achievement as an important area for further intervention research, particularly regarding the potential value of sustained academic support for socioeconomically disadvantaged students.

Second, the positive associations observed for Academic Engagement and Self-Efficacy, Academic Self-Concept, and Parental Educational Expectations suggest that psychosocial and family-related factors are relevant correlates of mathematics achievement and may represent important targets for further educational research. These findings do not establish that modifying these factors would causally improve achievement; however, they are consistent with prior evidence emphasizing the potential importance of students’ confidence, persistence, engagement, and beliefs about their academic competence. Similarly, the positive association with parental educational expectations highlights the relevance of family educational contexts and supportive academic routines for students’ learning experiences (Katsantonis, 2025; Qin et al., 2026).

Third, the negative associations involving School Violence and Peer Aggression, School Violence Climate, and Perceived Discrimination at School indicate that the psychosocial quality of the school environment is meaningfully related to mathematics achievement. Safe, orderly, and inclusive schools may provide emotional and relational conditions that are supportive of students’ engagement in challenging academic tasks. These findings do not demonstrate that reducing violence or discrimination will necessarily improve mathematics achievement; however, they are consistent with the broader educational relevance of promoting safer and more inclusive school environments. Policies and interventions addressing school violence and discrimination may therefore warrant evaluation not only for their socioemotional and disciplinary outcomes, but also for their potential academic implications.

Finally, the observed differences in longitudinal mathematics achievement change across socioeconomic and sex groups suggest that equity-oriented educational approaches should consider the joint configuration of multiple social positions rather than focusing on a single dimension in isolation. However, because the additional moderation involving Indigenous background was sensitive to the functional specification of SES, the present findings do not support strong policy conclusions for specific Indigenous subgroups. Instead, they highlight the importance of culturally responsive and context-sensitive educational practices, particularly in settings where socioeconomic disadvantage, sex-related inequalities, and Indigenous background intersect. Future intervention and policy research should evaluate whether such approaches are effective in reducing educational disparities.

### 4.6. Strengths, Limitations, and Future Research

This study has several strengths. First, it used a large national longitudinal cohort of Chilean students, allowing mathematics achievement change to be examined across a four-year period between Grade 4 and Grade 8. Second, the study incorporated predictors from multiple ecological domains, including individual psychosocial factors, family resources, school climate factors, and contextual characteristics. Third, the three-level mixed-effects modeling strategy accounted for repeated measurements within students and clustering of students within their Grade 4 schools, while effect-size estimates, predicted marginal means, simple-effect analyses, and SES sensitivity analyses provided complementary evidence regarding the robustness and substantive magnitude of the findings. Finally, the joint examination of Time, SES, Sex, and Indigenous Background allowed heterogeneity in longitudinal mathematics achievement change to be evaluated from an intersectional perspective, while also showing that the higher-order moderation involving Indigenous background was sensitive to the functional specification of SES.

Despite these strengths, several limitations should be acknowledged. First, although the longitudinal design allowed us to examine change in mathematics achievement over time, the analyses were based on observational data and therefore do not support causal interpretations. The associations identified in this study should be understood as evidence of relationships among psychosocial, family, school climate, contextual factors, and mathematics achievement, rather than as causal effects. In particular, the baseline psychosocial, family, and school-climate predictors were modeled as time-invariant main effects and therefore should be interpreted as correlates of achievement level across the two assessment occasions, not as determinants of longitudinal change.

Second, an additional limitation concerns selective longitudinal attrition and the use of complete-case analysis. Students who were retained longitudinally had substantially higher Grade 4 mathematics achievement than those who were not retained (M = 265.48 vs. 237.31; Cohen’s d = 0.60), indicating that attrition was not random with respect to baseline achievement. Among students who were observed at both waves, complete-case inclusion was associated with a smaller baseline achievement difference (M = 268.01 vs. 259.14; d = 0.19), while sociodemographic differences were comparatively small. These selection processes may limit the representativeness of the analytical sample and may have influenced the magnitude of the estimated longitudinal differences. However, the direction of this potential bias cannot be determined from the present analyses.

Another limitation is that most explanatory variables were measured at baseline in Grade 4. This approach allowed us to examine how early individual, family, school, and contextual characteristics were associated with mathematics achievement across the two assessment occasions, but it did not capture changes in these factors between Grade 4 and Grade 8. Moreover, because several psychosocial predictors, such as academic self-concept and engagement, were measured concurrently with Grade 4 mathematics achievement, their associations with the baseline score include a cross-sectional component. Future research could incorporate time-varying predictors to examine how changes in academic self-beliefs, family resources, school climate, discrimination, or student motivation are associated with subsequent mathematics achievement change.

In addition, the questionnaire-based measures involve measurement limitations. For the multi-item ordinal scales, internal consistency was estimated using ordinal Cronbach’s alpha and McDonald’s omega based on polychoric correlation matrices, yielding coefficients ranging from 0.77 to 0.95 for alpha and from 0.77 to 0.95 for omega. These results indicate adequate to very high internal consistency across the multi-item measures. However, internal consistency alone does not constitute comprehensive psychometric validation. Evidence regarding dimensionality, construct validity, and external validation would be required to more fully establish the measurement properties of these indices. In addition, some family and contextual indicators were based on one or two items, for which internal consistency coefficients were not reported, limiting the extent to which complex constructs could be represented. Accordingly, these measures should be interpreted as secondary-data indicators with varying levels of psychometric information rather than as fully validated standalone scales. This distinction is particularly important because the present study relied on secondary SIMCE questionnaire data and did not independently revalidate the latent structure of each construct.

A further limitation is that the study included two measurement occasions. Although this design allowed the estimation of achievement change between Grade 4 and Grade 8, additional waves would make it possible to model the shape of longitudinal change, including potential nonlinear patterns, and identify more specific periods in which achievement gaps widen or narrow. Future studies could extend this approach by incorporating additional assessment points across primary and secondary education and by estimating more flexible growth models when sufficient repeated measurements are available.

The representation of school context also warrants consideration. School-level clustering was defined using students’ Grade 4 school membership because the school-climate and contextual predictors were measured at baseline. Consequently, the model accounted for clustering within the baseline school but did not capture possible school changes between Grade 4 and Grade 8 or changes in school-level conditions across the observed period. Future studies with longitudinal school-membership information could examine whether school mobility and changing school contexts contribute to mathematics achievement change.

SES was modeled as an ordinal covariate to estimate the socioeconomic gradient in mathematics achievement change. This specification was parsimonious and theoretically aligned with the ordered nature of the SES measure, while allowing SES to be incorporated as a continuous adjustment variable in the mixed models. However, this approach assumes a linear socioeconomic gradient and may not capture possible threshold effects or nonlinear differences between SES categories. To examine the robustness of this assumption, a sensitivity analysis was conducted with SES modeled categorically. The main Time × SES and Time × Sex × SES patterns remained supported, indicating that the central socioeconomic and socioeconomic-by-sex findings were robust to the functional specification of SES. In contrast, the four-way Time × Sex × Indigenous Background × SES interaction was not statistically supported under the categorical specification, indicating that the additional moderation involving Indigenous background was sensitive to how SES was modeled. Future research could extend this analysis using more granular socioeconomic indicators or flexible nonlinear specifications, particularly for intersectional subgroup analyses.

Finally, Indigenous background was operationalized as a dichotomous indicator. Specifically, the secondary SIMCE data identified whether the student’s father and/or mother was reported as belonging to one of the Indigenous peoples recognized in Chile, using yes/no response categories for each parent. This measure therefore captures a broad indicator of Indigenous family background rather than the student’s own Indigenous identity. Although this measure was appropriate for the available data, it does not capture the diversity of Indigenous peoples, cultural identities, language experiences, or degrees of cultural affiliation in Chile. Future research should incorporate more nuanced measures of Indigenous identity and culturally relevant educational experiences to better understand how sociocultural recognition and exclusion shape academic experiences and longitudinal achievement change.

Overall, this study contributes to the literature on mathematics achievement change by showing that achievement levels and longitudinal differences between Grade 4 and Grade 8 are associated with psychosocial, family, school climate, socioeconomic, and sociocultural factors. The findings suggest that educational inequalities are not static but may widen across the observed period, particularly across socioeconomic groups and in ways that vary by sex. Evidence for additional differentiation by Indigenous background was more sensitive to model specification and should therefore be interpreted cautiously. Understanding these dynamics is important for informing future research and the development and evaluation of educational policies and interventions aimed at promoting more equitable mathematics learning opportunities across primary and lower secondary education.

## 5. Conclusions

This study showed that mathematics achievement between Grade 4 and Grade 8 among Chilean students was associated with predictors operating across psychosocial, family, school climate, socioeconomic, and sociocultural domains. The findings indicate that socioeconomic inequalities in mathematics achievement were not only present in primary education but became larger across the observed period. Students from higher socioeconomic groups showed more favorable longitudinal achievement change, whereas students from the lowest socioeconomic group showed a slight decline or only limited improvement, depending on subgroup characteristics.

The final model further demonstrated that longitudinal mathematics achievement change differed across socioeconomic and sex groups, while additional differentiation involving Indigenous background was more sensitive to the functional specification of SES. This result supports a psychosocial, ecological, and intersectional understanding of mathematics achievement, suggesting that academic development cannot be fully understood by examining socioeconomic, demographic, or sociocultural characteristics separately.

These findings highlight the importance of continued attention to socioeconomic disparities and to the psychosocial, family, and school-context factors associated with mathematics achievement. They also identify potentially relevant areas for future intervention and policy research, including students’ academic engagement and self-beliefs, family educational expectations, and safe, inclusive, and culturally responsive school environments. Because the present study was observational, the effectiveness of interventions targeting these dimensions should be established through future evaluative research.

## Figures and Tables

**Figure 1:**
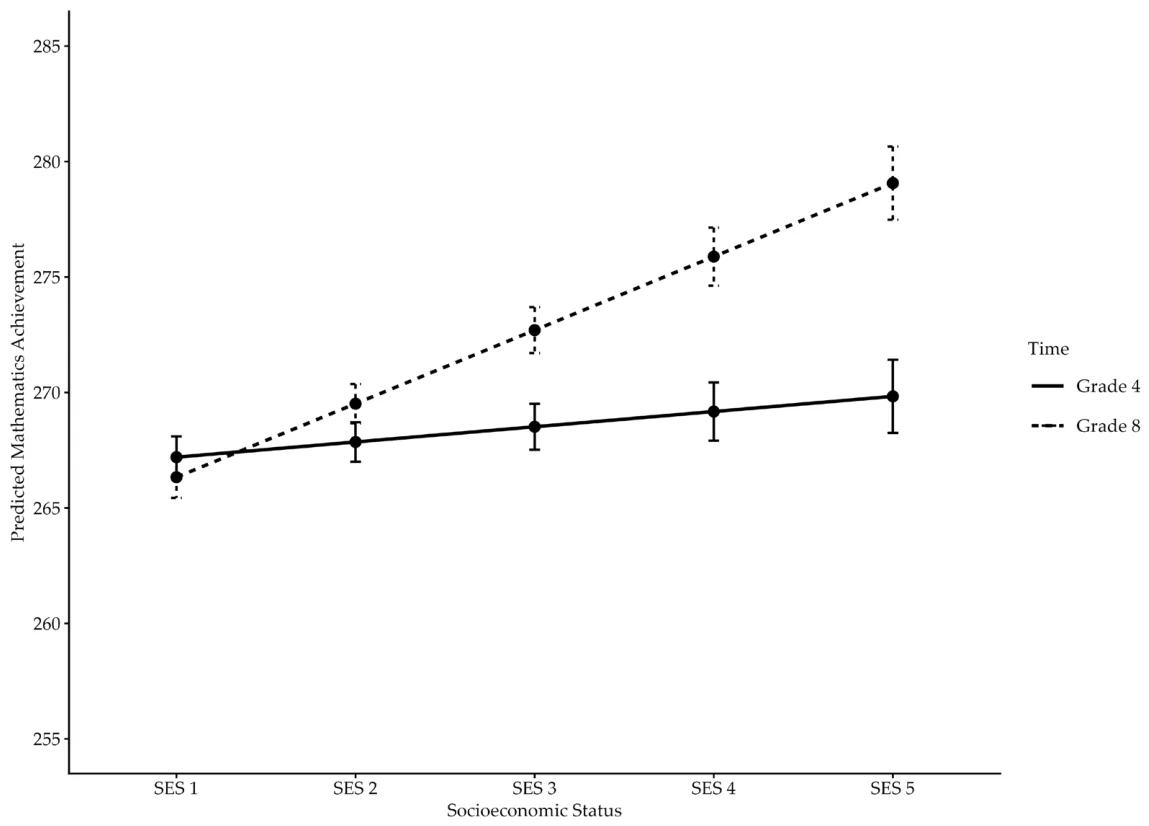
Predicted longitudinal mathematics achievement change from Grade 4 to Grade 8 according to socioeconomic status. Note. Predicted marginal means were derived from the final REML three-level linear mixed model adjusted for individual psychosocial, family, school climate, and contextual characteristics. SES = socioeconomic status.

**Figure 2:**
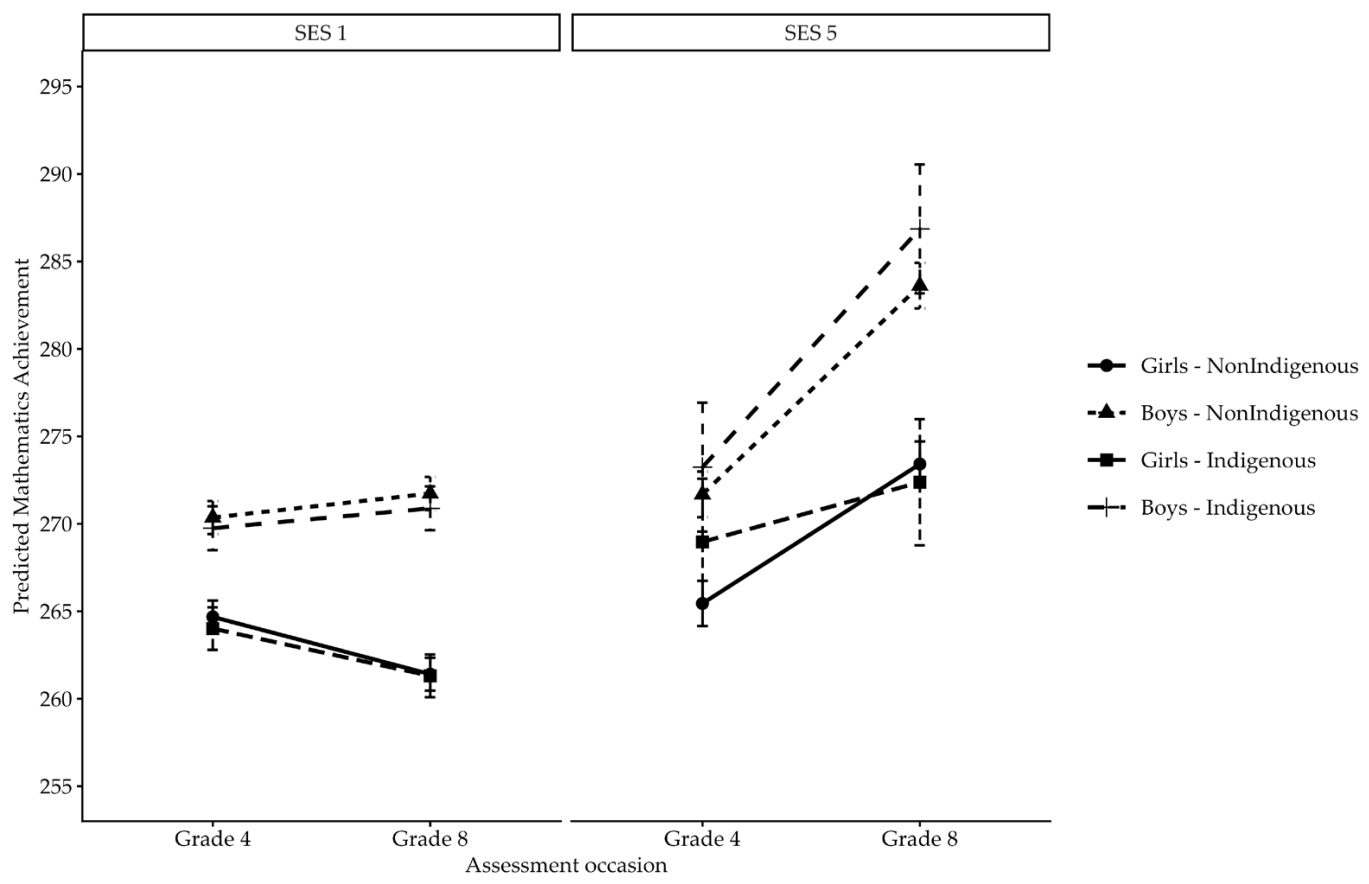
Predicted longitudinal mathematics achievement change from Grade 4 to Grade 8 by sex and Indigenous background at SES 1 and SES 5. Note. Predicted marginal means were derived from the final REML three-level linear mixed model adjusted for individual psychosocial, family, school climate, and contextual characteristics. SES = socioeconomic status. Error bars represent 95% confidence intervals.

**Table 1:** Characteristics of the Analytical Sample (N = 121,109).

**Panel A. Continuous Variables**	**M**	**SD**	**Min**	**Max**
Mathematics Achievement (Grade 4)	268.0	46.2	93.7	374.7
Mathematics Achievement (Grade 8)	269.5	48.9	131.2	404.9
Academic Engagement and Self-Efficacy	3.11	0.39	1.0	4.0
Academic Self-Concept	2.92	0.53	1.0	4.0
Teacher Regulation and Normative Guidance	3.51	0.53	1.0	4.0
School Safety Perception	3.03	0.83	1.0	4.0
School Violence and Peer Aggression	2.00	0.70	1.0	4.0
Parental Education	2.40	0.90	1.0	5.0
Cultural Capital (Books at Home)	3.00	1.00	1.0	5.0
Parental Educational Expectations	4.90	1.00	1.0	6.0
Perceived Discrimination at School	1.20	0.40	1.0	4.0
Classroom Order and Engagement	3.50	0.40	1.0	4.0
School Violence Climate	1.70	0.50	1.0	4.0
Teacher Collaboration	3.20	1.00	1.0	5.0
Student Misconduct	1.90	0.60	1.0	4.0
**Panel B. Categorical Variables**				
Variable	*n*		%	
Boys	58,236		48.1	
Girls	62,873		51.9	
Indigenous students	18,530		15.3	
Non-Indigenous students	102,579		84.7	
Public schools	40,476		33.4	
Subsidized private schools	69,789		57.6	
Private paid schools	10,844		9.0	
Urban schools	108,886		89.9	
Rural schools	12,223		10.1	

Note. All questionnaire-based predictors and sociodemographic characteristics were measured at baseline. Higher scores on questionnaire-based indices indicate higher levels of the corresponding construct.

**Table 2:** Comparison of three-level linear mixed models for mathematics achievement change.

Model	−2 Log Likelihood	AIC	BIC
Null model	2,445,779	2,445,787	2,445,828
Integrated model	2,423,654	2,423,702	2,423,951
Time × Sex	2,423,193	2,423,243	2,423,503
Time × Indigenous Background	2,423,629	2,423,679	2,423,939
Time × Socioeconomic Status	2,422,672	2,422,722	2,422,982
Time × Sex × Indigenous Background	2,423,169	2,423,225	2,423,516
Time × Sex × Indigenous Background × SES_c	**2,422,212**	**2,422,282**	**2,422,646**

Note. Models were estimated using maximum likelihood (ML) estimation with random intercepts for students and Grade 4 schools. Lower AIC and BIC values indicate better model fit. SES_c was included as an ordinal covariate centered at the middle category. Under the primary linear SES specification, the four-way interaction model was retained as the final model. Bold values indicate the fit statistics of the retained final model.

**Table 3:** Selected fixed-effect estimates from the final three-level linear mixed model of mathematics achievement change (N = 121,109).

Predictor	B	St(B)	SE	df	t	*p*	95% CI
Intercept	278.20	—	1.13	5903	247.05	<0.001	[276, 280.4]
Time (Grade 8)	2.35	—	0.21	121,101	11.43	<0.001	[1.9, 2.8]
Boys vs. girls	5.96	—	0.32	178,247	18.60	<0.001	[5.3, 6.6]
Public school	−26.10	—	1.07	6111	−24.43	<0.001	[−28.2, −24.0]
Subsidized private school	−17.69	—	1.01	5455	−17.53	<0.001	[−19.7, −15.7]
Urban school	2.94	—	0.63	7969	4.66	<0.001	[1.7, 4.2]
Socioeconomic status (SES_c)	0.19	—	0.18	155,114	1.08	0.278	[−0.16, 0.54]
Time × Boys	4.30	—	0.29	121,101	14.65	<0.001	[3.7, 4.9]
Time × Indigenous	−2.00	—	0.83	121,101	−2.41	0.016	[−3.6, −0.4]
Time × SES_c	2.81	—	0.13	121,101	22.19	<0.001	[2.56, 3.06]
Time × Boys × Indigenous	2.72	—	1.18	121,101	2.30	0.022	[0.4, 5.0]
Time × Indigenous × SES_c	−1.28	—	0.48	121,101	−2.68	0.007	[−2.22, −0.35]
Time × Boys × Indigenous × SES_c	1.76	—	0.69	121,101	2.57	0.010	[0.42, 3.11]
**Individual Psychosocial Factors**							
Academic Engagement and Self-Efficacy	15.32	0.13	0.29	119,009	52.51	<0.001	[14.75, 15.89]
Academic Self-Concept	8.36	0.09	0.21	118,290	39.89	<0.001	[7.95, 8.77]
Teacher Regulation and Normative Guidance	3.03	0.03	0.20	119,065	14.99	<0.001	[2.63, 3.43]
School Safety Perception	0.32	0.006	0.14	118,278	2.34	0.019	[0.05, 0.59]
School Violence and Peer Aggression	−6.44	−0.09	0.17	120,376	−38.62	<0.001	[−6.77, −6.11]
**Family Resources**							
Parental Education	4.10	0.08	0.17	120,413	24.12	<0.001	[3.77, 4.44]
Cultural Capital (Books at Home)	2.48	0.05	0.12	118,958	21.23	<0.001	[2.25, 2.71]
Parental Educational Expectations	4.85	0.10	0.12	119,766	40.33	<0.001	[4.61, 5.08]
Perceived Discrimination at School	−7.12	−0.05	0.28	119,324	−25.25	<0.001	[−7.68, −6.57]
**School Climate Factors**							
Classroom Order and Engagement	5.13	0.04	0.48	35,906	10.75	<0.001	[4.20, 6.07]
School Violence Climate	−1.66	−0.02	0.46	35,182	−3.63	<0.001	[−2.56, −0.76]
Teacher Collaboration	−0.30	−0.007	0.16	31,285	−1.92	0.054	[−0.60, 0.01]
Student Misconduct	0.25	0.003	0.34	39,423	0.74	0.462	[−0.42, 0.92]

Note. The final model was estimated using restricted maximum likelihood (REML) with random intercepts for students and Grade 4 schools. B = unstandardized estimated coefficient; St(B) = Standardized coefficients; SE = standard error; df = Satterthwaite degrees of freedom; CI = confidence interval. Positive coefficients indicate higher estimated mathematics achievement scores, whereas negative coefficients indicate lower estimated mathematics achievement scores. Grade 4 was the reference category for Time. Reference categories were girls, non-Indigenous students, rural schools, and private paid schools. SES_c was included as an ordinal covariate centered at the middle socioeconomic category. All questionnaire-based predictors were measured at baseline in Grade 4 and continuous predictors were mean-centered prior to model estimation. For terms involved in interactions, coefficients are conditional contrasts relative to the reference categories and to SES_c = 0. For parsimony, the table reports the main fixed effects and selected conditional interaction estimates relevant to the retained four-way interaction structure. Non-displayed lower-order interaction terms were retained in the model to preserve the hierarchical structure. Type III tests reported in the text provide the omnibus tests of fixed effects and interaction terms.

## Data Availability

The datasets analyzed in this study are not publicly available due to confidentiality restrictions. Requests to access the datasets should be directed to the Chilean Education Quality Assurance Agency statistical information portal: https://informacionestadistica.agenciaeducacion.cl/#/bases (accessed on 20 September 2026).

## Supplementary Materials

The following supporting information can be downloaded at https://www.mdpi.com/article/10.3390/bs16101748/s1, Table S1: Description and internal consistency of questionnaire-based indices included in the final mathematics model.
