# Supplementary material for: Socioeconomic and Intersectional Differences in Mathematics Achievement Change: Psychosocial and Contextual Evidence from Chilean Students
Source: Behav Sci (Basel). 2026 Sep 24;16(10):1748. doi: 10.3390/bs16101748 (PMC13701909; doi:10.3390/bs16101748)
Supplement: Supplementary file 1 [file behavsci-16-01748-s001.zip › behavsci-4456962-supplementary.pdf]

Supplementary Table S1

Description and internal consistency of questionnaire-based indices included in the final mathematics model.

**Variable/construct**	**Domain**	**Source/ questionnaire**	**Scale**	**N° items**	**(α,ω)**	**Interpretation**
Academic Engagement and Self-Efficacy	Individual	Student	Likert	9	(.77,.77)	Higher scores indicate stronger academic engagement and perceived self-efficacy.
Academic Self-Concept	Individual	Student	Likert	8	(.80,.80)	Higher scores indicate more positive academic self-concept.
Teacher Regulation and Normative Guidance	Individual/school experience	Student	Likert	5	(.83,.83)	Higher scores indicate stronger perceived teacher regulation, guidance, and classroom norms.
School Safety Perception	Individual/school experience	Student	Likert	2	_	Higher scores indicate greater perceived school safety.
School Violence and Peer Aggression	Individual/school experience	Student	Likert	10	(.93,.93)	Higher scores indicate greater perceived school violence and peer aggression.
Parental Education	Family	Parent/guardian	Ordinal	2	_	Higher scores indicate higher parental educational attainment.
Cultural Capital (Books at Home)	Family	Parent/guardian	Ordinal	1	_	Higher scores indicate a larger number of books at home.
Parental Educational Expectations	Family	Parent/guardian	Ordinal	1	_	Higher scores indicate higher parental educational expectations.
Perceived Discrimination at School	Family/contextual	Parent/guardian	Likert	10	(.95,.95)	Higher scores indicate greater perceived discrimination or exclusion at school.
Classroom Order and Engagement	School	Teacher	Likert	5	(.85,.85)	Higher scores indicate greater classroom order and student engagement.
School Violence Climate	School	Teacher	Likert	8	(.91,.91)	Higher scores indicate greater perceived school violence climate.
Teacher Collaboration	School	Teacher	Likert	5	(.91,.91)	Higher scores indicate greater teacher collaboration.
Student Misconduct	School	Teacher	Likert	4	(.89,.89)	Higher scores indicate more frequent student misconduct.

Note. For multi-item ordinal scales, internal consistency was estimated using ordinal Cronbach's alpha and McDonald's omega based on polychoric correlation matrices. Reliability coefficients were not reported for single-item or two-item indicators.
